# *In vitro* shared transcriptomic responses of *Aedes aegypti* to arboviral infections: example of dengue and Rift Valley fever viruses

**DOI:** 10.1186/s13071-020-04253-5

**Published:** 2020-08-05

**Authors:** Séverine Licciardi, Etienne Loire, Eric Cardinale, Marie Gislard, Emeric Dubois, Catherine Cêtre-Sossah

**Affiliations:** 1 CIRAD, UMR ASTRE, 97490 Sainte Clotilde, La Réunion, France; 2 ASTRE, University of Montpellier, CIRAD, INRAe, Montpellier, France; 3 CIRAD, UMR ASTRE, 34395 Montpellier, France; 4 MGX-Montpellier Genomix, IGF, INSERM, CNRS, University of Montpellier, Montpellier, France

**Keywords:** *In vitro*, Transcriptomic responses, *Aedes aegypti*, Dengue, Rift Valley fever

## Abstract

**Background:**

Arthropod borne virus infections are the cause of severe emerging diseases. Among the diseases due to arboviruses, dengue (DEN) and Rift Valley fever (RVF) are in the top ten in the list of diseases responsible of severe human cases worldwide. Understanding the effects of viral infection on gene expression in competent vectors is a challenge for the development of early diagnostic tools and may enable researchers and policy makers to better anticipate outbreaks in the next future.

**Methods:**

In this study, alterations in gene expression across the entire *Aedes aegypti* genome during infection with DENV and RVFV were investigated *in vitro* at two time points of infection, the early phase (24 h) and the late phase (6 days) of infection using the RNA sequencing approach

**Results:**

A total of 10 upregulated genes that share a similar expression profile during infection with both viruses at early and late phases of infection were identified. Family B and D clip-domain serine proteases (CLIP) were clearly overrepresented as well as C-type lectins and transferrin.

**Conclusions:**

Our data highlight the presence of 10 viral genes upregulated in *Ae. aegypti* during infection. They may also be targeted in the case of the development of broad-spectrum anti-viral diagnostic tools focusing the mosquito vectors rather than the mammalian hosts as they may predict the emergence of outbreaks.
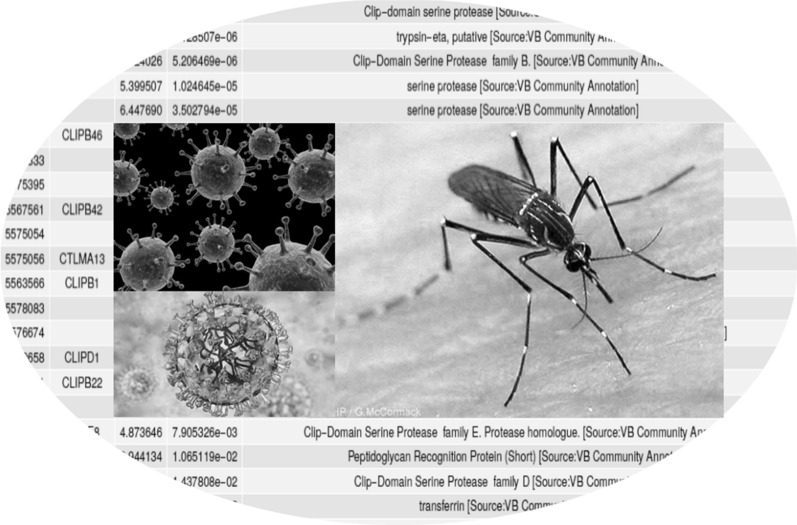

## Background

In the past decade, there has been a worldwide emergence of arboviruses-related diseases such as Zika, chikungunya, dengue, West Nile (WN) and Rift Valley fever (RVF) leading to a global health burden with important socioeconomic impacts. An acute systemic febrile illness that often includes headache, weakness, myalgia, joint pain, arthralgia, or gastrointestinal symptoms is commonly associated to these infections in their hosts with specific patterns such as encephalitis or meningitis for WN and retro-orbital pain for dengue and RVF (https://www.cdc.gov/westnile/dengue/riftvalleyfever/chikungunya). They are mostly transmitted by the same *Aedes* mosquito species except for WN where the *Culex* species have a major role in the transmission cycle [1].

Rift Valley fever virus (RVFV) is a category A priority zoonotic pathogen due to its potential to cause severe economic distress and major health issues (mass abortions and neonatal mortality in ruminants and human deaths) following infection by direct contact with infected animals (tissues and aerosols) or through the bites of infected mosquitoes. RVFV is widespread in sub-Saharan Africa and has expanded its geographic range to Egypt including the River Nile Delta, the Arabian Peninsula [2], the eastern Horn of Africa (Kenya, Uganda and Rwanda) and in the Indian Ocean zone (the Comoros archipelago and Madagascar) [2–5].

Dengue viruses are spread to people through the bites of infected *Aedes* species mosquitoes (*Aedes aegypti* or *Ae. albopictus*). About one in four people infected with dengue will get sick with symptoms that can be mild or severe depending on several parameters such as the serotype of dengue virus involved and the host immunity. Dengue virus (DENV) exists as four serotypes (DENV-1, -2, -3 and -4) that are phylogenetically related and loosely antigenically distinct [6]. For this reason, a person can be infected with a dengue virus as many as four times in his or her lifetime. In certain cases, it can progress to more complicated forms, such as dengue haemorrhagic fever (DHF) and dengue shock syndrome (DSS) with high mortality rates, as well as significant economic burdens [7]. Dengue is endemic in more than 120 countries in southeast Asia, the Americas, the western Pacific, Africa and the eastern Mediterranean regions [8].

The ongoing outbreaks of RVF occurring in Mayotte, a French island part of the Comoros archipelago at the same time than dengue [9, 10] as well as outbreaks of dengue in other islands of the Indian Ocean area (La Reunion and Seychelles islands) [11, 12] underline the need of a better control of both diseases by implementing early warning systems resulting from interactions between people, mosquitoes, arboviruses, and environmental factors. An integrated approach taking into account the host and the vector compartments involved in vector borne diseases may help in anticipating or preventing the spread of huge outbreaks through adequate control measures [13, 14]. Efforts have been made to better understand the mechanisms of pathogenesis of these viruses including pathways essential for replication and to develop innovating and appropriate diagnostic tools, therapeutics and vaccines [15–17]. Current diagnostic methods mainly developed for the detection of specific viral molecular signatures in the host compartment by molecular based techniques [18, 19] were adapted to trapped arthropods [20–23] with limitations such as (i) the need of high viral load being present in the vector at the time of the trapping, (ii) the time window in which the competent vectors exhibit a sufficient viral load, (iii) the specificity of the detection of one pathogen rather than having a larger detection system able to detect several arboviral infections at the same time. Applicability of other assays such as the Lawrence Livermore Microbial Detection Array (LLMDA) revealed the presence of mosquito-borne viruses and insect-specific viruses in field-collected mosquitoes with similar limitations [24]. Recently, in 2018, Fukutani et al. [25, 26] proposed an innovative approach, based on the transcriptomic response of the vectors to various arboviruses. In short, they listed a group of co-regulated genes whose expression levels significantly changed in vectors infected by different viruses.

Indeed, one could expect that native immunity related genes could be expressed in a non-pathogen-specific manner in response to infection. The identification of a set of 110 genes which behave as infection-specific, allowed unambiguous classification of infected and uninfected vectors. Interestingly, infection-specific expression patterns (either activation or repression) of genes have been identified to play important roles in immunity, stress and chemosensory reception. This approach is paving the way to surveillance methods that allow for a non-specific detection of arboviral diseases in vectors responsible of their transmission. Following this approach, our study was designed to search for upregulated genes in *Ae. aegypti* cells lines in response to two major arboviruses infections, dengue and Rift Valley fever virus infections by RNA sequencing.

Therefore, finding a diagnostic tool based on a set of markers as an early sign of viral infections in the vector component rather than the detection of putative pathogens transmitted by the vector itself, has to be seen as an accurate way to anticipate epizootics/epidemics simultaneously at a population and large-scale level.

## Methods

### Virus growth

The stock of the Smithburn RVFV strain (OBP, Onderstepoort Biological Products, Onderstepoort, South Africa) and the stock of serotype 2 human DENV supplied by Dr Jaffar-Bandjee (CHU La Réunion) for research purposes were produced on African green monkey kidney Vero cells using DMEM (Gibco Life Technologies, Illkirch, France) containing 10% foetal bovine serum (FBS) (Gibco Life Technologies), 2mM L-glutamine (Gibco Life Technologies). Virus-containing medium was harvested when the cytopathic effect (CPE) exceeded 75%, and the viral infectivity titre was determined by limiting dilution [27].

### Cell culture and viral infections

The CCL-125 *Ae. aegypti* derived cell line Aag2 (Pasteur Institute, Paris, France) [28] was used for infection studies. The cells were grown at 28 °C in Schneider’s *Drosophila* medium (Gibco Life Technologies) supplemented with 10% FBS (Gibco Life Technologies), 1% L-glutamine 1mM, 1% penicillin-streptomycin (Gibco Life Technologies). Aag2 cells (2 × 10^6^ cells/well in a 24-well format plate) were infected either at a multiplicity of infection (MOI) of 0.1 for RVFV or at a MOI of 1 for DENV based on the literature [29, 30]. Viruses were allowed to propagate for 24 h and 6 days to assess early and late genes expression. Three controls named Mock were included, (i) non-infected Aag2 cells to assess the expression of genes related to cellular functions (Mock A), (ii) double RVFV (MOI of 0.1) and DENV (MOI of 1) infected and UV inactivated cells to assess the expression of activated genes following the viral entry into the cells (Mock B), and (iii) non-infected Aag2 cells submitted to a heat shock through an incubation step at 37 °C for 30 min to assess the expression of genes involved in cellular oxidative stress (Mock C). Supernatants were removed and spun down, cells were harvested and stored at − 80 °C until use. All experimental infections were performed under biosafety level 3 (BSL-3) conditions. The experiment was undertaken independently in triplicate.

### RNA extraction

Total RNA was extracted from (i) DENV serotype 2 infected cell lines, (ii) RVFV infected *Ae. aegypti* cell lines and (iii) the three controls (Mock A, B and C) supernatants at days 1 and 6 using the NucleoSpin RNA virus kit (Macherey-Nagel, Hoerdt, France) according to the manufacturer’s instructions, except that the lysis buffer RAV1 was supplemented with linear acrylamide (Ambion, Life technologies, Illkirch, France) at a concentration of 5 mg/ml and Proteinase K (20 mg/ml) instead of the RNA carrier provided in the kit. The eluted RNA was submitted to a Turbo Dnase digestion (Ambion, Life Technologies) (2 units/µl) for 30 min at 37 °C, followed by inactivation and clean up steps performed with the RNeasy MinElute Cleanup kit (Qiagen, Les Ulis, France) and stored at − 80 °C until use. The quality of the isolated total RNA from each sample was checked using the Nano RNA chips in a 2100 Bioanalyzer (Agilent, Les Ulis, France) and quantitation was performed using NanoDrop 2000 (Thermo Fisher Scientific, Illkirch, France). RNA samples with RNA integrity number (RIN) ≥ 8 were selected for library preparation.

### qRT-PCR reactions

DENV and RVFV viral infections were confirmed by a previously described RT-qPCR [18, 31]. Briefly, for each sample, qPCR reactions were performed in triplicate using the AgPath One Step RT-PCR 2× Mix (Ambion, Life Technologies) in an Mx 3005P QPCR System™ (Stratagene, Agilent, Les Ulis, France).

### Library construction for RNA sequencing

Libraries were constructed using the Truseq stranded mRNA sample prep kit (ref. RS-122-2101; Illumina, San Diego, USA) according to the manufacturer's instructions (https://www.illumina.com/products/by-type/sequencing-kits/library-prep-kits/truseq-stranded-mrna.html#productLongDescription). Briefly, poly-A RNAs were purified using oligo-d(T) magnetic beads. The poly-A+ RNAs were fragmented and reverse transcribed using random hexamers, Super Script II (ref. 18064-014; Life Technologies, Les Ulis, France) and Actinomycin D. During the second strand generation step, dUTP substitued dTTP. This prevents the second strand to be used as a matrix during the final PCR amplification.

Double stranded cDNAs were adenylated at their 3’ ends before ligation was performed using Illumina’s indexed adapters. Ligated cDNAs were amplified following 15 cycles PCR and PCR products were purified using AMPure XP Beads (ref. A63881; Beckman Coulter Genomics, Villepinte, France). Libraries were validated using a Fragment Analyzer (Agilent) and quantified using the KAPA Library quantification kit (ref. KK4824; Roche, Boulogne-Billancourt, France).

Equimolar pools of 9 libraries were constituted and sequencing was performed on a HiSeq2500 (Illumina, San Diego, USA) using the single read protocol (50 nt) on 4 lanes.

### Bioinformatics analysis

The quality of the data was assessed using FastQC from the Babraham Institute. Potential contaminants were investigated with the FastQ Screen software [32] from the Babraham Institute. RNA-seq 50 nt reads were aligned to the *Ae. aegypti* assembly (AaegL5.0) with a set of gene model annotations (GCF_002204515.2_AaegL5.0_genomic.gff downloaded from NCBI on 10 July 2018), using the splice junction mapper TopHat v2.1.1 [33], which used bowtie 2.2.8 [34]. Final read alignments having more than 3 mismatches were discarded. Gene counting was performed using HTSeq-count [35] version 0.9.0 (union mode). Since data come from a strand-specific assay, the read has to be mapped to the opposite strand of the gene. Before statistical analysis, genes with less than 15 reads (cumulating all the analysed samples) were filtered out. Dataset was then transformed in log CPM (counts per million) and a trimmed mean of M-values normalization was used to correct for libraries size effect. Description of the samples are present in https://github.com/loire/CCS_RNAseq_analysis. A multidimensional scaling (MDS) was used to represent the bray-curtis distances calculated between all replicates and samples on the 100 most expressed genes.

### Statistical analysis

The following steps were followed: (i) fit a quasi-likelihood negative binomial generalized log-linear model to count data; genewise statistical tests were conducted for a given coefficient or contrast (glmQLfit) (quasi-likelihood (QL) F-test against the FC threshold); (ii) genewise statistical tests were conducted for a given coefficient or contrast relative to a specified fold-change threshold (here a 2-fold change in expression threshold) (function glmTreat); (iii) differentially expressed (DE) genes were identified using the Bioconductor [36] package *edgeR* 3.20.1 [37]. Data were normalized using the relative log expression (RLE) [38] normalization factors. Genes with adjusted *P*-value less than 5% (according to the FDR, Benjamini-Hochberg method) were declared differentially expressed.

A list of candidate genes was obtained as the intersection between the lists of DE genes of each of the DENV and RVFV viral treatments. The CPM of each gene candidate was then plotted for each sample for visual inspection. Raw count data and an R script generating all results and figures are available at https://github.com/loire/CCS_RNAseq_analysis/tree/master.

## Results

### High quality of mRNA sequence dataset generated

To obtain a broad picture of the host response to DENV and RVFV infections, RNA-Seq was used to analyze differential gene expression at the mRNA level. The mRNA was isolated and purified from Mock, RVFV and DENV infected cells at 1 and 6 days post-infection to assess for early and late detection genes expression.

High-throughput sequencing generated an average of 11,021,430 million reads per sample. Approximately 94.63% of the reads were mapped to the *Ae. aegypti* genome (GCF_002204515.2_AaegL5.0_genomic.gff, [39]) and there were anywhere between 7028 and 9565 genes expressed per replicate at each time point. A total of 9580 genes expressed above 0.5 count per million in at least 3 samples were subsequently analyzed.

### Dataset exploration

**Fig. 1:**
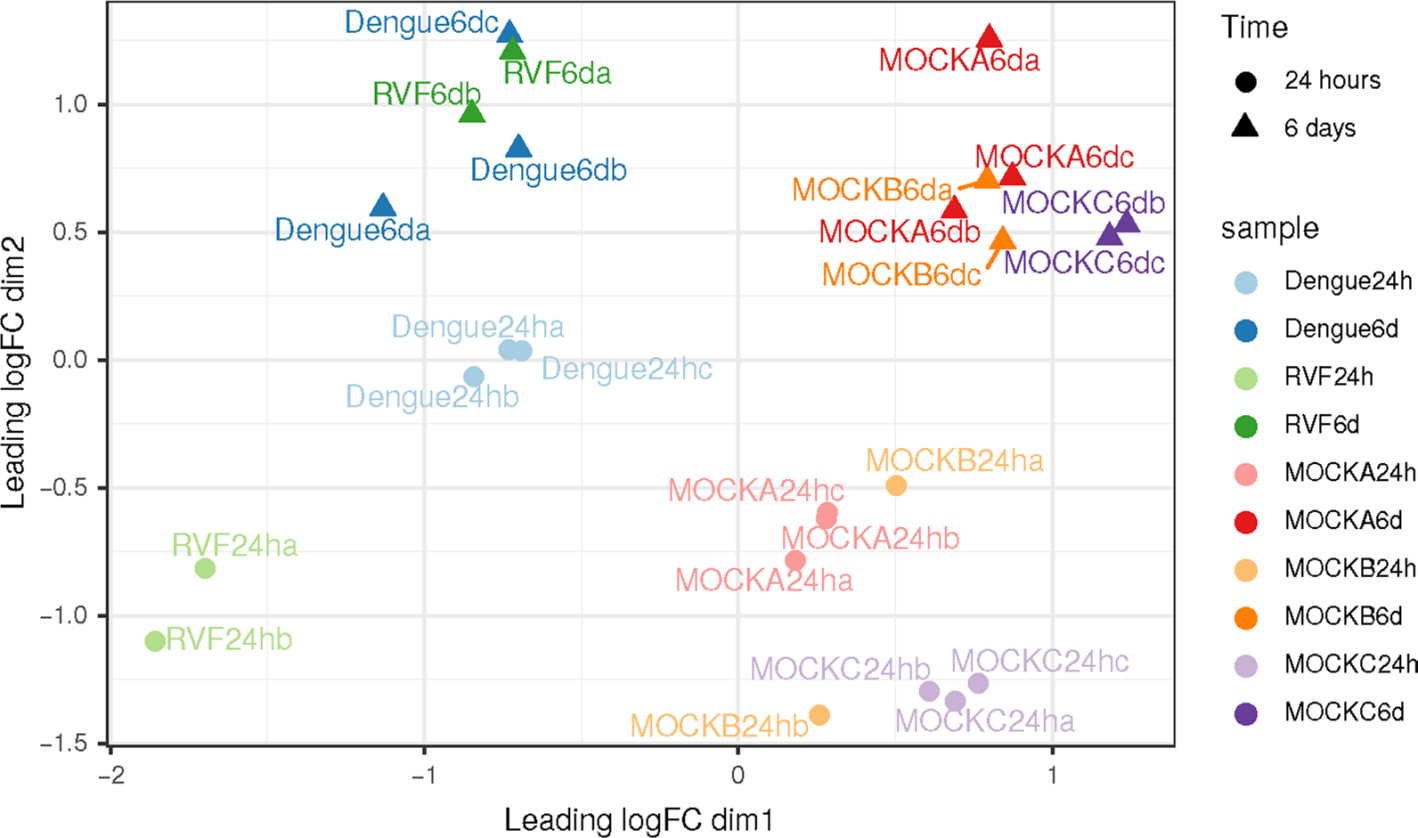
Non-parametric multidimensional scaling (MDS) plot of filtered samples. Each point represents a sample. Color represents the type of sample (RVF: Rift Valley fever). Mocks: negative controls of infections (see main text for description). Time post-infection is represented with shapes of different color intensity: clear circle for early responses (24 h); opaque triangles for late responses (6 days or 144 h). Points clustered in space share a similar expression pattern on a subset of 100 highly expressed genes. Fold changes in gene expression are projected on two dimensions (axes). The first axis separates viral and mock samples, the second axis separates early (24 h) and late (6 days) responses

Selected samples exhibit a good homogeneity among samples after normalization. A nice dataset, with replicates well grouped and a net separation of groups of samples was observed. One of the RVF replicate at 24 h post-infection was discarded due to its failure to the quality control check (library size < 5 millions of reads) (Fig. 1).

The first dimension separates mock infection from viral infection, and the second dimension separates early (24 h post-infection) and late (6 days post-infection) responses. Additionally, late responses to viral and mock infections are similar as well as early responses to viral and mock infections, indicating the possibility to conduct a direct comparison between them to search for common differential expression of genes in response to both viruses.

### Differential expression analysis

#### Early viral response

Expression values obtained at 24 h post-infection in viral infected samples (DENV and RVFV) were compared to mock samples. A total of 27 genes have been found to be upregulated at the early phase. Upregulated genes detected in viral infections are, for the most part, related to native immune defense mechanisms. Family B and D clip-domain serine proteases (CLIP) are clearly over-represented with 10 genes (CLIP-B1, CLIP-B15, CLIP-B22, CLIP-B34, CLIP-B35, CLIP-B42, CLIP-B46, CLIP-D1, CLIP-D6 and CLIP-E8) belonging to this family out of the 15 total expressed genes prohibitin, a strongly conserved and ubiquitously expressed protein in eukaryotic cells, C-type lectins (CTLMA-13 and CTLMA-14), transferrin (Tf1), peptidoglycan recognition protein (PGRP) and Gram-negative binding protein (GNBP) have been also identified as upregulated genes (Table 1). Some of these genes were found to be upregulated only at the early phase of infection such as Niemann-Pick type C family genes, macroglobulin/complement and serine protease inhibitor (SRPN3) (Table 1).

**Table 1:** Early response upregulated genes detected in viral infections

NCBI gene ID	Gene name	logFC	FDR	Gene description (Source)
5563663	CLIPB35	3.06	1.12E-12	Clip-Domain Serine Protease family B (VB Community Annotation)
5564201	CLIPB15	4.14	5.37E-11	Clip-Domain Serine Protease family B (VB Community Annotation)
5564288	CTLMA14	4.01	4.20E-09	C-Type Lectin (CTL) - mannose binding (VB Community Annotation)
5578692		2.58	3.67E-07	Clip-domain serine protease (UniProtKB/TrEMBL; Acc: Q1HQI3)
5570115		7.1	5.13E-06	Trypsin-eta, putative (VB Community Annotation)
5563616	CLIPB34	5.52	5.21E-06	Clip-Domain Serine Protease family B (VB Community Annotation)
5574170		5.4	1.02E-05	Serine protease (VB Community Annotation)
5575350		6.45	3.50E-05	Serine protease (VB Community Annotation)
5565977	CLIPB46	3.61	4.70E-05	Clip-Domain Serine Protease family B (VB Community Annotation)
5572333		6.15	6.90E-05	Clip-domain serine protease, putative (VB Community Annotation)
5575395		3.03	7.53E-04	Prohibitin, putative (VB Community Annotation)
5567561	CLIPB42	3.23	2.09E-03	Clip-Domain Serine Protease family B (VB Community Annotation)
5575054		4.59	2.09E-03	Serine protease, putative (VB Community Annotation)
5575056	CTLMA13	6.06	2.32E-03	C-Type Lectin (CTL) - mannose binding (VB Community Annotation)
5563566	CLIPB1	2.94	2.37E-03	Clip-Domain Serine Protease family B (VB Community Annotation)
5578083		2.71	4.11E-03	F-spondin (VB Community Annotation)
5576674		3.55	5.22E-03	ATP-binding cassette sub-family A member 3, putative (VB Community Annotation)
5569658	CLIPD1	4.9	6.46E-03	Clip-Domain Serine Protease family D (VB Community Annotation)
5570931	CLIPB22	4.09	7.35E-03	Clip-Domain Serine Protease family B (VB Community Annotation)
5578380		2.24	7.47E-03	Bm-40 precursor (VB Community Annotation)
5567077	CLIPE8	4.87	7.91E-03	Clip-Domain Serine Protease family E. Protease homologue (VB Community Annotation)
5571998	PGRPS1	3.94	1.07E-02	Peptidoglycan Recognition Protein (short) (VB Community Annotation)
5573598	CLIPD6	3.27	1.44E-02	Clip-Domain Serine Protease family D (VB Community Annotation)
5579417	Tf1	4.8	1.74E-02	Transferrin (VB Community Annotation)
5569420	GNBPA1	6.63	1.87E-02	Gram-Negative Binding Protein (GNBP) or Beta-1 3-Glucan Binding Protein (BGBP) (VB Community Annotation)
5572428		4	3.56E-02	Macroglobulin/complement (VB Community Annotation)
5564141		3.21	4.92E-02	Niemann-Pick Type C-2, putative (VB Community Annotation)

*Abbreviations*: FC, fold change; FDR, false discovery rate corrected *P*-value; VB, vector base (https://www.vectorbase.org/)

#### Late viral response

A total of 22 genes were found to be significantly upregulated in the late response. Very interestingly, 10 upregulated genes are common between the early and the late responses, 6 days post-infection, relative to the control: 5 of the 10 Clip-domain serine proteases (CLIP-B15, CLIP-B34, CLIP-B35, CLIP-B46 and CLIP-D1), transferrin (Tf1), C-type lectin (CTLMA-13 and CTLMA-14), PGRP and GNBP. Some genes have been found to be upregulated only at the late phase of infection such as macroglobulin/complement, serine protease inhibitor, C-type lysozyme (LYSC11) and the 40S ribosomal protein S2 (RpS2) (Table 2).

**Table 2:** Late response upregulated genes detected in viral infections

Gene ID	Gene name	logFC	FDR	Gene description
110676293	LYSC11	2.91	1.35E-10	C-Type Lysozyme (Lys-A) (VB Community Annotation)
5563663	CLIPB35	2.89	2.50E-10	Clip-Domain Serine Protease family B (VB Community Annotation)
5564288	CTLMA14	4.07	9.29E-09	C-Type Lectin (CTL) - mannose binding (VB Community Annotation)
5565977	CLIPB46	5.05	1.50E-08	Clip-Domain Serine Protease family B (VB Community Annotation)
5564201	CLIPB15	3.09	3.16E-06	Clip-Domain Serine Protease family B (VB Community Annotation)
5574112		3.42	5.84E-05	GTP cyclohydrolase i (VB Community Annotation)
5575350		6.38	6.41E-05	Sserine protease (VB Community Annotation)
5574170		4.21	7.38E-05	Serine protease (VB Community Annotation)
5566832	SRPN3	7.02	1.66E-04	Serine Protease Inhibitor (serpin) likely cleavage at T/I (VB Community Annotation)
5578692		2.29	5.05E-04	Clip-domain serine protease (UniProtKB/TrEMBL; Acc: Q1HQI3)
5572333		6.2	6.33E-04	Clip-domain serine protease, putative (VB Community Annotation)
5563725		4.67	1.50E-03	Serine protease inhibitor, serpin (VB Community Annotation)
5572968	RpS2	2.35	2.74E-03	40S ribosomal protein S2 (UniProtKB/TrEMBL; Acc: Q1HRV1)
5569658	CLIPD1	4.81	6.39E-03	Clip-Domain Serine Protease family D (VB Community Annotation)
5574952		4.8	6.58E-03	Metalloproteinase, putative (VB Community Annotation)
5576150		3.55	7.79E-03	Lipase 1 precursor (VB Community Annotation)
5572428		5.11	7.79E-03	Macroglobulin/complement (VB Community Annotation)
5563616	CLIPB34	3.39	7.90E-03	Clip-Domain Serine Protease family B (VB Community Annotation)
5569420	GNBPA1	6.98	8.71E-03	Gram-Negative Binding Protein (GNBP) or Beta-1 3-Glucan Binding Protein (BGBP) (VB Community Annotation)
5579417	Tf1	6.46	1.12E-02	Transferrin (VB Community Annotation)
5571998	PGRPS1	4.88	3.36E-02	Peptidoglycan Recognition Protein (short) (VB Community Annotation)
5575056	CTLMA13	4.31	4.11E-02	C-Type Lectin (CTL) - mannose binding (VB Community Annotation)

*Abbreviations*: FC, fold change; FDR, false discovery rate corrected *P*-value; VB, vector base (https://www.vectorbase.org/)

#### Level of expression of upregulated genes (early and late)

**Fig. 2:**
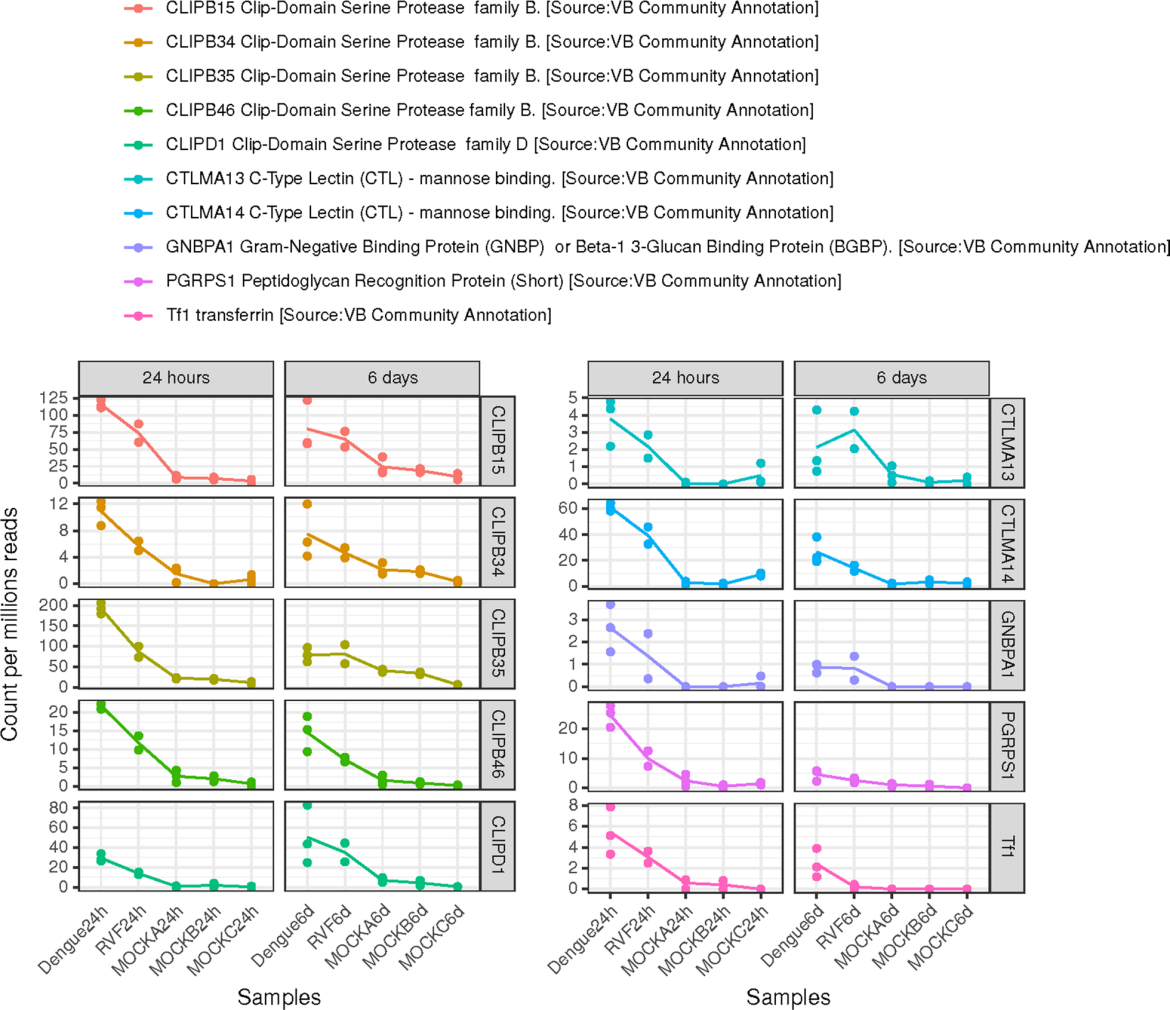
Counts per million in each sample was used to check for actual overexpression in samples infected by a virus. Each plot shows the mean expression level of a gene (expressed as count per million reads in the samples) as well as the values obtained for each of the replicates

Genes upregulated in viral *versus* mock infection, at both late and early stages of infection, were assessed for their intersection. Known genes that were filtered are presented Fig. 2. Counts per million in each sample is used to check for actual overexpression in samples infected by a virus.

#### Late versus early response

Only two genes were found to be significantly upregulated between early and late response: F-spondin and nidogen. The latter is also related to viral infection [40] but was not upregulated when compared to mock infections in our data.

## Discussion

Mosquitoes have been shown worldwide as vectors of several viral diseases of great importance not only for public health, but also for animal health. The current techniques used to detect virus circulation in the vectors include virus isolation, detection of RVF specific viral nucleic acids by conventional and real-time reverse transcriptase polymerase chain reaction (RT-PCR), in the entire body or in the saliva collected on FTA cards of field caught specimens as well as vector competence studies on cage-raised mosquito specimens [19, 20, 41–45]. All of these techniques are focusing on one pathogen at a time. Whole genome sequencing and annotation of the Zika, chikungunya, RVF and dengue vector, *Ae. aegypti*, has enabled a comparative phylogenomic analysis of the insect immune repertoire allowing deeper understanding of insect immune systems. Mosquito innate immunity is now able to recognize and respond to a huge number of pathogens, in a dynamic game where either host or pathogen is the winner reflecting in part continuous re-adjustment between accommodation and rejection of pathogens [46]. Evolving features have been associated with different functional gene categories including several host factors regulated during mosquito viral infection.

The main goal of our study was to identify upregulated genes in cell lines of *Ae. aegypti* in response to two major arboviruses infections, dengue and RVF infections by RNA sequencing.

Our comparative analysis highlighted the expression profile of 39 significantly upregulated genes following either early (27), late (22) or both early/late (10) indicating a potentially conserved transcriptomic signature of dengue and RVF emerging infections. Among annotated genes, family B and family D clip-domain SPs (CLIP-B15, CLIP-B34, CLIP-B35, CLIP-B46 and CLIP-D1), C-type lectins-mannose binding (CTLMA) namely CTLMA-13 and CTLMA-14 and transferrin were largely represented in the early/late viral upregulated response we obtained. Peptidoglycan recognition protein (PGRP) and Gram-negative binding protein (GNBP) were also identified as upregulated genes following both viral infections at both early and late stages. Data supporting NTU discovery points out the need for continuous amendments of the reference genome annotation [47]. Chymotrypsin-related SPs form a large family of enzymes that hydrolyze peptide bonds at different rates and with various degrees of specificity [48, 49]. SPs and their serine protease homologs (SPHs) have been previously described to participate in digestion, defense, development, and other physiological processes [50]. Like human clotting factors, they form complex networks to stop bleeding and fight infection. In each insect species with a known genome, SP-related proteins form a large family with 60–400 members [51–53]. In mosquitoes, numbers of clip-domain SPs/SPHs genes named CLIPs identified in genomes are 63 in *Ae. aegypti*, 55 in *Anopheles gambiae* and 45 in *Drosophila melanogaster* [46, 54]. They have been investigated for possible roles in antiparasitic responses and are known to regulate several invertebrate defense responses, including hemolymph coagulation, antimicrobial peptide synthesis, and melanization of pathogen surfaces [51–56]. Transferrin is also well known to be involved in viral responses [57, 58]. The C-type lectins (CTLs), have been implicated in immunity as opsonins and modulators of melanization. Prohibitin upregulated at the early stage of infection is described as a highly conserved and ubiquitously expressed protein in eukaryotic cells [59]. Niemann-Pick type C family and macroglobulin/complement genes have been already shown as related to dengue infections [60, 61].

Our data highlight the presence of upregulated viral gene targets in the mosquito during infection. These preliminary findings, specifically the ten genes found to be upregulated and common between the early and late phase of infection, have to be confirmed *in vivo* on artificially RVFV and DENV infected adult *Ae. aegypti* mosquito populations and in field-collected mosquitoes in an epizootic context of RVFV and/or DENV infections. Although early detection of DENV or RVFV circulation in the vector component of the epidemiological cycle will be carried out on mosquito species known to be competent for both viruses, this does not allow us to conclude on the proven or potential vector role for each of these viruses tested.

The development of two separate molecular based diagnostic tools, each of them able to detect five of the ten identified upregulated genes (CLIP-domain SP families, transferrin, prohibitin, C-type lectins, Fig. 2) at the same time by a multiplex PCR approach is the next step. Experiments involving a DENV and RVFV mouse model to show that virally upregulated genes have an impact on the viral replicative cycles could also be performed.

## Conclusions

To the best of our knowledge, this is the first study to compare two major arboviral infections, RVF and dengue that may occur in the same area and at the same time, as it is the case now in the Comoros archipelago, specifically on the island of Mayotte. Investigating the effects of DENV and RVFV *in vitro* infections in the mosquito was the first-step. The detection of ten upregulated genes that could be combined into a set of two mutliplex-PCR reactions has been highlighted and must be checked *in vivo* through (i) experimentally infected native populations of *Ae. aegypti*, and (ii) field caught specimens, which should help to anticipate the occurrence of outbreaks.

## Data Availability

The dataset supporting the conclusions of this article is available in a github repository (https://github.com/loire/CCS_RNAseq_analysis, https://github.com/loire/CCS_RNAseq_analysis/blob/master/DE_results.xlsx), along with scripts and instructions for complete reproducibility of the analysis.

## Abbreviations

DENV: dengue virus
WN: West Nile
RVF: Rift Valley fever
DHF: dengue haemorrhagic fever
DSS: dengue shock syndrome
CTLMA: C-type lectins-mannose binding
CLIP: clip-domain serine protease
